# The Therapeutic Potential of tRNA-derived Small RNAs in Neurodegenerative Disorders

**DOI:** 10.14336/AD.2021.0903

**Published:** 2022-04-01

**Authors:** Haihua Tian, Zhenyu Hu, Chuang Wang

**Affiliations:** ^1^Ningbo Key Laboratory of Behavioral Neuroscience, Ningbo University School of Medicine, Ningbo, Zhejiang, China.; ^2^Zhejiang Provincial Key Laboratory of Pathophysiology, School of Medicine, Ningbo University, Ningbo, Zhejiang, China.; ^3^Department of Physiology and Pharmacology, Ningbo University School of Medicine, Ningbo, Zhejiang, China.; ^4^Department of Laboratory Medicine, Ningbo Kangning Hospital, Ningbo, Zhejiang, China.; ^5^Department of Child Psychiatry, Ningbo Kanning Hospital, Ningbo, Zhejiang, China

**Keywords:** Amyotrophic lateral sclerosis, Alzheimer’s disease;, neurodegenerative diseases, Parkinson’s disease;, pontocerebellar hypoplasia, tRNA-derived small RNAs

## Abstract

Gene expressions and functions at various levels, namely post-transcriptional, transcriptional, and epigenetic, can be regulated by *transfer* RNA (tRNA)-derived small RNAs (tsRNAs), which are as well-established as tRNA fragments or tRFs. This regulation occurs when tsRNAs are created through the special endonuclease-mediated cleavage of mature or precursor tRNAs. However, tsRNAs are newly discovered entities, and molecular functions associated with tsRNAs are still not clearly understood. There is increasingly robust evidence suggesting that specific tsRNAs perform fundamental tasks in the pathogenesis of neurodevelopmental, neurodegenerative, and neurobehavioral disorders. Indeed, the patterns of tsRNA expression are uncertain and could be altered in patients suffering from Parkinson’s disease, pontocerebellar hypoplasia, amyotrophic lateral sclerosis, Alzheimer’s disease, and other neurodegenerative disorders. In the present article, a review is conducted of recent domestic and international progress in research on the potential cellular and molecular mechanisms of tsRNA biogenesis. We also describe endogenous tsRNAs during neuronal development and neurodegenerative disorders, thereby providing theoretical support and guidance for further revealing the therapeutic potential of tsRNAs in neurodegenerative disorders.

## 1.Introduction

tRNA, or transfer RNA, is a well-characterized non-coding RNA that serves as a key translational mediator [1] and modulates a range of non-translational processes in cells. Specific endonucleases such as angiogenin (ANG) and Dicer cleave pre- or mature tRNAs under specific stress circumstances and yield tRNA-derived small RNAs (tsRNAs, also termed tRNA-derived stress-induced RNAs; tiRNAs or tRNA-derived fragments; tRFs) in a cell type-dependent behavior [2]. The functions of tsRNAs as principal regulators of physiological or pathological processes have not been highlighted in preliminary investigations. Nevertheless, extensive evidence has described tsRNAs in various conditions, such as viral infection, metabolic disorders, stroke, cancer, and neurological disorders [3-5]. The human nervous system is extremely complicated and comprises of diverse regulatory pathways that regulate neurobiological functions. Over the recent decades, the role of tsRNAs in the development, disorder, and function of the central nervous system (CNS) has drawn the attention of a considerable number of researchers in this field. Neurodegenerative diseases (NDs) are conditions that result in the progressive deterioration of myelin in the CNS, ultimately leading to neuronal dysfunction [6]. Specific neuronal subsets within the CNS play critical roles in processes such as movement, sensory information processing, and decision-making. Since neurons mainly lack a regenerative capacity, they are considerably susceptible to irreversible damages. The prevalence of NDs is increasing as the aging population increases worldwide. Furthermore, the prolonged and indolent course of these types of diseases can create severe burdens for patients, their families, and the society at large. Very recently, the role and behavior of tsRNAs in the onset and progression of NDs have been thoroughly investigated and well-characterized. In the next sections, the classification of tsRNAs and their roles in NDs will be comprehensively discussed (As shown in Table 1).

**Table 1:** List of selected tsRNAs involved in NDs with their molecular mechanism.

tsRNAs	ND	Type	Type of experiment	Mechanism	Expression level	Refs.
AS-tDR-011389	AD	i-tRF	Profiling in mouse model	Regulation of LTP	Down	[61]
AS-tDR-013428	AD	tRF-5	Profiling in mouse model	Regulation of Aβ	Down	[61]
AS-tDR-011775	PD	tRF-1	Profiling in mouse model	Regulation of Mobp, Park2 to determining the morphology of axons in neurons	Up	[61]
AS-tDR-005058	PD	i-tRF	Profiling in mouse model	Regulated through the Rab6ip2 /ERC1/CAST2/ELKS and presynaptic active zone protein interaction	Up	[61]
tiRNA-Tyr	AD	5’tiRNA	Prefrontal lobe cortex samples of AD patients by small RNA sequencing	Enhance the vulnerability to oxidative stress on neurons	Down	[63]
tiRNA-Arg	AD	5’tiRNA	Prefrontal lobe cortex samples of AD patients by small RNA sequencing	Involved in synapse formation in AD	Down	[63]
tRF5-GlyGCC	AD	tRF-5	Hippocampus of AD patients	Unknown	Up	[62]
tRF5-GluCTC	AD	tRF-5	Hippocampus of AD patients	Unknown	Up	[62]
tRF5-GlyCCC-2	AD	tRF-5	Hippocampus of AD patients	Unknown	Up	[62]
tRF5-ProAGG	AD	tRF-5	Hippocampus of AD patients	Interact with ribosomes and polysomes lead to global translation inhibition and upregulation of a specific low molecular weight peptidy1-tRNA product	Up	[62]
tiRNA^Cys^	ALS	5’tiRNA	Eukaryotic cells	Inhibit translation initiation by displacing eIF4F from cap structures (m7G)	Up	[37]
tiRNA^Ala^	ALS	5’ tiRNA	Eukaryotic cells	Inhibit translation initiation by displacing eIF4F from cap structures (m7G) and induce the assembly of stress granules (SGs)	Up	[37]
tiRNA-ValCAC	ALS	5’tiRNA	Mouse models of ALS, ALS patients	Inhibit protein translation	Up	[80]
Several tRFs	PD	tRNA-dervied fragments	Prefrontal cortex samples, cerebrospinal fluid (CSF), serum samples from PD patients	Unknown	Up	[68]

## 2.tsRNA classification and biogenesis

**Figure 1.**
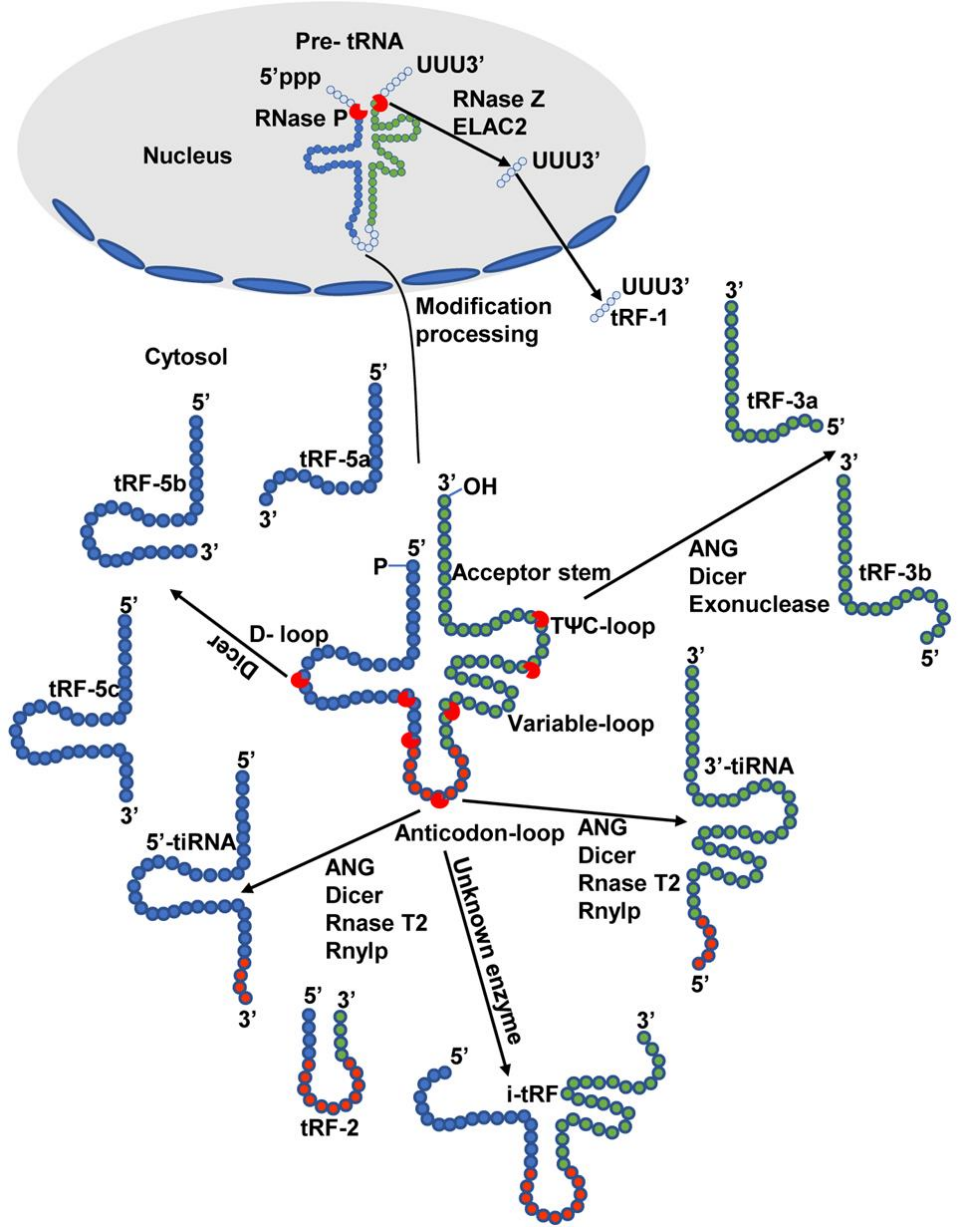
Principal categorization and biogenesis of tsRNAs. tsRNAs can be categorized into different subtypes including tiRNAs and tRFs. tRFs are divided into five sub-categories, namely i-tRF, tRF-5, tRF-3, tRF-2, and tRF-1. tRF-5, tRF-2, and i-tRF originates from mature tRNAs digested through Dicer, Angiogenin (ANG), or other RNase at various sites, while tRF-1 originates from pre-tRNA digested through RNase Z. tiRNAs are classified into two main subtypes, 3’-tiRNA and 5’-tiRNA, which originate from the mature tRNAs cleaved *via* ANG at the anticodon rings. Based on the cleavage sites and length of the tRNAs, different colors represent different types. All processing enzymes involved are indicated in the figure.

Many investigations have aimed to classify tsRNAs into different subtypes, including tRFs and tiRNAs [7]. The convention of specific naming for these tsRNAs is based on the positions at which mature or parental pre-tRNAs are cleaved. The biogenesis and fundamental classifications of tsRNAs are demonstrated in Figure 1. tiRNAs are 29-50 nucleotides (nt)-long tsRNAs generated *via* specific mature tRNA anticodon loop cleavage under stress circumstances, including hypoxia, viral infection, heat shock, amino acid starvation, or ultraviolet radiation. tRFS are 14-30 nt-long tsRNAs derived from mature or pre-tRNAs [8]. These tiRNAs are, in turn, categorized into 3’- and 5’-tiRNAs based on whether the 3’ or 5’ sequence, respectively, harbors the anticodon cut locus extending from the corresponding mature tRNA ends to the anticodon loop [9]. Furthermore, tRFs are assorted into tRF-5, tRF-3, tRF-2, tRF-1, and i-tRF subtypes based on their individual cleavage sites (Fig. 1). Among these subtypes, i-tRFs primarily originate from internal sites within mature tRNAs, and their names are selected based on the starting point of the 5’ end of the tRNA. A-tRF, V-tRF and i-tRF subtypes correspond to fragments generated through the anticodon ring and variable region cleavage, whereas D-stem cleavage yields D-tRFs [10]. tRF-1 members are generated via ELAC2- or RNase Z-mediated cleavages of precursor 3’-tRF sequences derived from precursor tRNA 3’-UTR sequences [11]. In contrast, tRF-2 members are derived from tRNA anticodon loop sequences in hypoxic contexts and lack 5’ or 3’ structures [12]. tRF-3 members are derived from 3’ mature tRNA ends following Dicer-, ANG-, or exonuclease-mediated TψC-loop cleavage. Fundamentally, tRF-3 tails contain “CCA” trinucleotide motifs derived from mature tRNA 3’ ends. The primary tRF-3 members are tRF-3b and tRF-3a, both of which are 18-22 nt long [13]. Moreover, tRF-5 is produced from 5’ mature tRNA ends via Dicer-mediated cleavage at the D-loop region. This yields tRF-5 members <30 nt; these can be sub-divided into tRF-5c (28-30 nt), tRF-5b (22-24 nt), and tRF-5a (14-16 nt) categories [13]. Among these, tRF-5c and tRF-5b are generated via anticodon stem and D-stem cleavage, while tRF-5a is generated via D-loop cleavage. Other types of tRNA fragments have been explored by taking advantage of high-performance sequencing, suggesting that tiRNAs and tRFs are more diverse than initially thought.

### 2.1. Key enzymes involved in tsRNA biogenesis

Several enzymes are essential for tiRNA maturation, including RNase Z, ELAC2, Dicer, and ANG [14, 15]. The endonuclease RNase Z is crucial for the 3’ maturation of tRNAs [16]. RNase Z processes tRNAs and tRFs, thereby maintaining a homeostatic balance between these substrates [17]. RNase Z cleaves tRNAs to give rise to tRF-1 [18]. Moreover, Dicer-dependent tRF processing has been divulged in human investigations [19]. For instance, Dicer expression suppresses the expression of certain tRNA^Gln^-derived tRFs [20], and similar findings have been observed in the context of Dicer1-mediated biogenesis in mature B cells [19]. Other studies have reported that the generation of certain 22-30 nt-long 5’ and 3’ tRFs is Dicer-independent in human cells, *Drosophila*, and *Schizosaccharomyces pombe*, indicating that the specific task of Dicer in tRF biogenesis is tissue- or cell type-dependent. Numerous surveys have demonstrated that ANG is closely related to the creation of smaller (22-30 nt) 5’ and 3’ tRFs and tiRNAs in HEK293 cells [21]. ANG secretion is enhanced under hypoxic conditions in tumor cell lines, wherein it plays a key role in stress responses [22]. Nevertheless, further *in vivo* research is warranted to elucidate the performance of ANG in the biogenesis of tsRNA.

### 2.2 Association between tRNA modification and tsRNA biogenesis

As key regulators of cellular processes, tRNAs must maintain normal structural stability and functional integrity, which are controlled by various post-transcriptional modifications [23]. Most of these modifications impact the anticodon loop region, with tRNA methylation influencing the ability of these RNAs to interact with proteins and ensure coding fidelity. Furthermore, these modifications affect tsRNA biogenesis. For example, Trm9 is a tRNA-methyltransferase that catalyzes the 5-methylcarbo-xylmethyl modification of uridine in yeast to reduce the susceptibility of tRNA molecules to get cleaved [24]. Similarly, the 5-methylcytosine modification of tRNA anticodon loop regions mediated by Dnmt2 prevents ANG-mediated cleavage of this region and thus reduces tiRNA levels [25]. Emerging evidence suggests that these modifications regulate a range of pathophysiological processes in diseases affecting the CNS or muscular tissues. Changes in tRNA abundance, modification, and aminoacylation levels influence mRNA decoding in a cell type-specific manner and potentially impact neurodegeneration. These tRNAs also interact with proteins in diverse contexts unrelated to translation, thereby indirectly influencing this process within neurons.

## 3.tsRNA regulatory mechanisms

**Figure 2.**
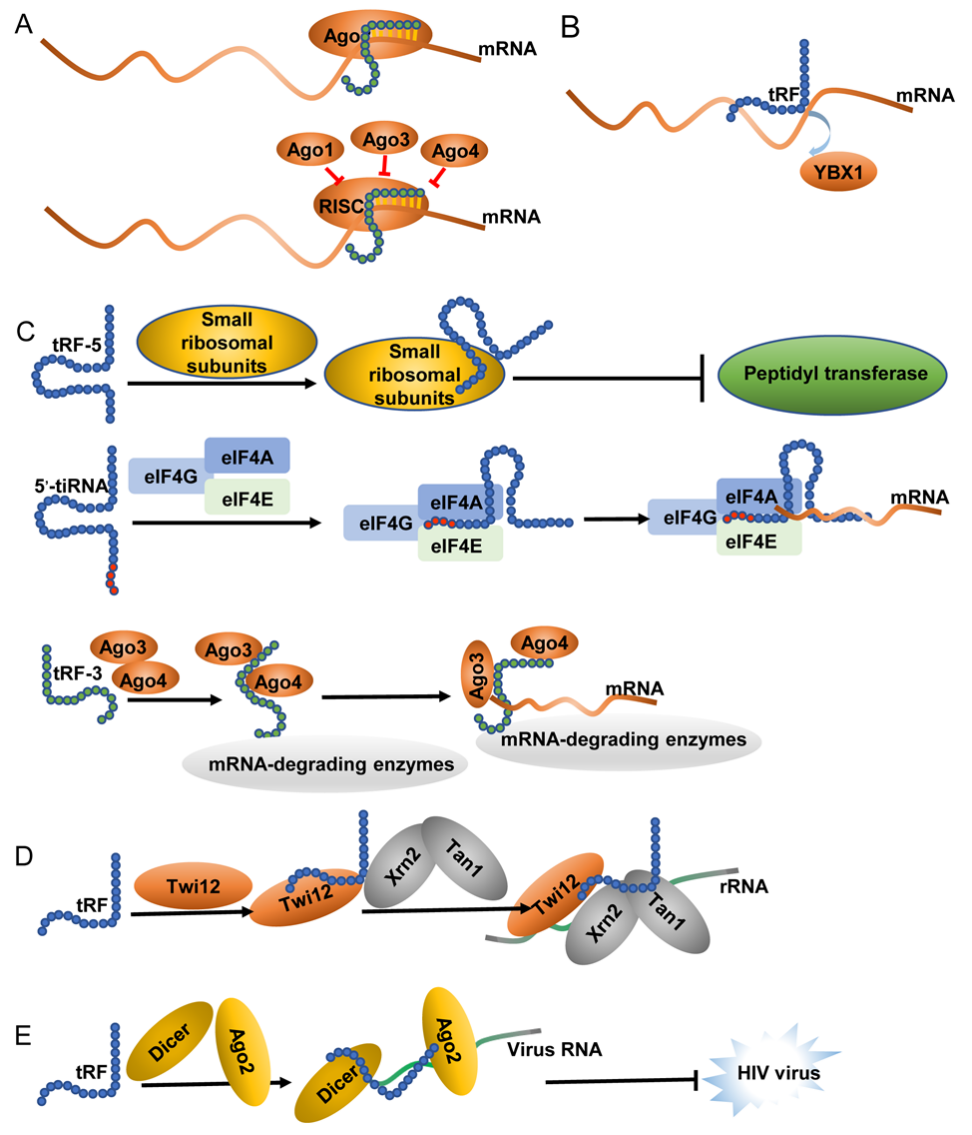
Mechanisms of action of tsRNAs. (A) miRNA-like performances in the regulation of gene expression; (B) tRF-mediated sequestration of RNA-binding proteins; (C) Regulation of protein translation; (D) tsRNA-mediated regulation of rRNA synthesis; (E) Regulation of RNA reverse transcription.

Although the functional roles of most tsRNAs are yet to be clarified, mounting evidence suggests that they can bind mRNAs (such as miRNAs or piRNAs), thereby regulating translation, controlling ribosome biogenesis, and sequestering key RNA-binding proteins (As shown in Fig. 2).

### 3.1. miRNA- and piRNA-like gene expression regulation

Several tsRNAs regulate gene expressions in a manner analogous to miRNAs and piRNAs. Indeed, certain tsRNAs are similar in length to miRNAs that bind argonaute (AGO) proteins via comparable functional mechanisms [26]. For instance, 17 nt-long tRNA^Leu^-derived fragments bolster the functionality of cancer stem cells in colorectal cancer and promote disease progression by targeting the 3’-untranslated regions (UTRs) of JAG2, thereby suppressing its expression [27]. Other tRFs and associated target mRNAs are associated with proteins related to the RNA-induced silencing complex (RISC), including GW182/TNRC6 and AGO [26, 28]. The 22-nt tRF-3072b derived from tRNA^Gly^ binds to AGO and suppresses RPA1 expression in B cells [19]. Furthermore, tRF-3001, tRF-3003, and tRF-3009 suppress the expression of specific mRNAs in cells that overexpress the parental tRNAs from which these tRFs are derived [28]. Moreover, the interaction of specific proteins with PIWI proteins and their gene silencing behavior have been demonstrated. For instance, RNA^Glu^-derived tRF-5c interacts with PIWIL4 to regulate *IL-4* expression and downregulate the expression of CD1A in human monocytes via sequence complementarity [29]. The ability of ts-3676 and ts-4521 to interact with PIWIL2 and AGO1/2 has also been reported [30], while ts-101 and ts-53 bind to piRNA-like PIWIL2 to regulate target gene expression [31].

### 3.2. tRF-mediated sequestration of RNA-binding proteins

Upon interaction with RNA-binding proteins (RBPs), the sequestration of these proteins could take place through tRFs mechanisms and will prevent them from binding to other RNA substrates. A range of i-tRFs originating from tRNA^Glu^, tRNA^Tyr^, tRNA^Asp^, and tRNA^Gly^ can interact with RBP YBX1, which is a mediator of cytoplasmic mRNA stability, thereby preventing its interaction with the 3’-UTR of oncogene transcripts in human breast cancer cells [4]. Similarly, tRF-5Gln binds to and sequesters IGF2BP1, preventing it from binding to the mRNA encoding the *c-Myc* oncogene, thus reducing its stability [32]. The RBP lupus autoantigen (La or SSB) stabilizes RNA polymerase III transcripts, including certain viral RNAs and pre-tRNAs. Interaction with tRF-1s sequesters this RBP and prevents it from binding to viral RNAs, thereby preventing viruses from hijacking this RBP to influence gene expression [33].

### 3.3. Regulation of protein translation

tsRNAs serve as both positive and negative regulators of translational activity in particular contexts. Seminal reports revealed the ability of tiRNAs to displace the eIF4F complex from mRNAs, thereby inhibiting translation [34]. The 5’ halves of tRNA^Ala^ and tRNA^Cys^ inhibit translation by binding to YBX1, thereby promoting stress granule (SG) formation [35]. Although the binding between these tsRNAs and YBX1 is essential for incorporating specific mRNAs into SGs, it is not required for translational suppression [36]. The two aforementioned forms of tsRNAs include terminal oligoguanine (TOG) motifs and form the structures of G-quadruplexes that are essential for SG functionality and translation inhibition [37, 38]. Other short tRF-5s, such as tRF-5^Gln^, have also been identified as mediators of translational repression. tRF-5^Gln^ is 19 nt in length and facilitates sequence-independent translational repression by interacting with a complex containing multiple aminoacyl tRNA synthetase (AARS) proteins [39]. Additionally, tRF-5s derived from tRNA^Ala^, tRNA^Cys^, and tRNA^Val^, which are 18 nt long, bind to the translation initiation factor PABPC1, thereby inhibiting translation in a manner dependent on PUS-7-dependent ψ8 modification [40]. tRFs promote translational activity in certain settings. For example, tRNA^Leu^-derived tRF-3011b, which is 22 nt in length, pairs with the mRNA of ribosomal proteins RPS15 and RPS28 to promote their respective translations [41]. In *Trypanosoma brucei*, the 3’ half of tRNAThr interacts with ribosomes to promote protein translation [42].

### 3.4. tsRNA-mediated regulation of rRNA synthesis

Ribosomes are essential mediators of mRNA translation. Ribosome biogenesis from pre-rRNAs is a tightly regulated process [43]. tsRNAs regulate rRNA biogenesis by functioning as members of the splicing complex of pre-rRNA (TXT) in protozoa (*Tetrahymena*) [44]. The TXT complex contains a 3-tRF that binds to the exonucleases, Xrn2 and Twi12. Following this binding, the stabilization and localization of Twi12 occur, which facilitates its exonuclease activity and cleavage of pre-rRNAs to regulate the synthesis of mature rRNAs [45].

### 3.5. Regulation of RNA reverse transcription

A subset of tsRNAs can suppress reverse transcription in the context of viral infections. For instance, Yeung et al. discovered a tRF that was 18 nt in length and expressed at high levels by the human immunodeficiency virus (HIV). By hybridizing with the 3’-terminus of human tRNA^Lys^, HIV viral RNA yields a dsRNA construct that, by viral reverse transcriptase, could be reversely transcribed to produce cDNA. The tRFs derived from this cDNA can, in turn, bind to Dicer and AGO2, thereby suppressing HIV viral RNA [46]. Host cell-derived tRF-3019 can initiate reverse transcription and promote viral reproduction due to the sequence complementarity between this tRF and the HTLV-1 primer binding site [47]. Infection of cells with a respiratory syncytial virus (RSV) promotes the production of ANG mediated tiRNAs. These tiRNAs serve as primers that initiate and enhance reverse transcription activity, thereby augmenting the ability of this virus to reproduce and infect other cells [48, 49]. These studies emphasize the ability of tsRNAs to regulate viral reverse transcription, highlighting these non-coding RNAs as potential targets that may aid in viral control.

### 3.6. Other tsRNA-related regulatory mechanisms

In addition to the mechanisms discussed so far, tsRNAs regulate various procedures. For example, specific tsRNAs suppress target gene expression to influence the proliferation and migration of cells [50] and regulate the transcription and binding of DNA-specific proximal RNA polymerase II. Additionally, tsRNAs can drive or suppress apoptosis. For example, 3’-tsRNA-LeuCAG promotes the apoptotic death of rapidly dividing cells both in a murine model of patient-derived orthotopic HCC and *in vitro* [41]. Other tsRNAs bind to apoptotic protease activating factor 1 (APAF1) and cytochrome C to create apoptotic bodies [51]. During hyperosmotic stress, ANG mediates competitive 3’-tiRNA and 5’-tiRNA binding to cytochrome C, resulting in the formation of ribonucleoprotein complexes, thereby inhibiting apoptotic body formation and enhancing cell survival [51, 52]. The encapsulation of tsRNAs within exosomes can also facilitate communication between cells [53]. tsRNAs regulates the epigenetic inheritance of patrilineally transmitted metabolic diseases [54], and T cell activation is similarly regulated by these tRNA-derived non-coding RNAs [55].

## 4.tsRNAs as regulators of neurodegeneration

Extensive evidence supports an association of abnormal tRNA metabolism and mutations in enzymes related to tRNA processing with the incidence of neurodegeneration [3, 56-58]. Notably, mutations affecting components of the tRNA splicing endonuclease complexes are thought to be linked to lower spinal motor neuron disorders. RNA kinase CLP1 is associated with tRNA splicing, and neurons derived from patients with mutations in the *CLP1* gene exhibit lower levels of mature tRNAs and unspliced pre-tRNA accumulation. These tsRNAs increase the motor neuron sensitivity to oxidative stress, indicating that non-coding RNAs may play a role in regulating neuronal redox homeostasis in non-pathological settings [3]. A missense mutation in the *CLP1* gene has been detected in patients suffering from microcephaly, cortical dysgenesis, and severe sensorimotor deficits; further, these symptoms are associated with abnormal tRNA splicing[3, 56, 57]. In some cases, *CLP1* mutations result in the generation of a tRF that increases neuronal sensitivity to p53-mediated oxidative damages, thereby leading to scarce familial NDs [56]. *CLP1* knockout in mice results in progressive motor neuron loss, correlated with tRNA Try and tRNA Arg-derived tsRNA accumulation [3, 56]. According to several investigations, four independent pedigrees were considered for the founding mutation in *CLP1* [3, 56-58]. Such impaired tRNA processing leads to the agglomeration of many tsRNAs and other fragments that can, in turn, contribute to the onset of NDs. Understanding the mechanisms underlying tRF processing disorders and neurodegeneration may lead to a better understanding of the etiology of these complex disorders and highlight novel approaches for patient management and treatment.

Several tsRNAs serve as important regulators of cellular function in the context of specific NDs in which they are dysregulated [59]. Indeed, altered tsRNA metabolism is linked to multiple neurodegenerative conditions, although more research is necessary to elucidate the diagnostic and prognostic value of these biomolecules. Below, we discuss several examples of tsRNAs and their relationships to specific NDs.

### 4.1. tsRNAs in Alzheimer’s disease (AD)

AD is the most pervasive and widely studied ND affecting the CNS [60]. The symptoms of this disease, which include memory impairments and progressive language difficulties, arise because of the progressive deterioration of the hippocampus and subcortical structures [60]. Patients with AD present with deposits of intracellular neurofibrillary tangles containing hyperphosphorylated tau protein alongside β-amyloid (Aβ) peptide-based extracellular plaques.

Recent work has indicated that tsRNAs may regulate the expression of important disease-related genes closely tied to the formation of Aβ peptides [61-63]. For example, Zhang et al. reported that AS-tDR-0111389 interacted with the endogenous CaMKII inhibitor Camk2n1, thereby influencing synaptic CaMKII-NMDAR binding and LTP regulation. Moreover, AS-tDR-0111389 interacts with P2ry1, which encodes the receptor protein PzY1 that contributes to the astroglial network dysfunction in AD via the purinergic signaling modulation. Furthermore, AS-tDR013428 targeted Rpsa to facilitate neurotoxic Aβ peptide generation and internalization [61]. In a recent investigation, Wu et al. reported significant tRF dysregulation in the hippocampus of patients with AD [62]. Additionally, Zhang et al. further employed a small RNA sequencing approach that revealed the expression of tsRNA-Arg and tsRNA-Tyr in the prefrontal cortex of patients experiencing AD [63]. Notably, the expression of certain tRFs was dependent on the disease stage and patient age, and ANG expression was significantly upregulated in patients with AD, emphasizing its performance as a mediator of tRF dysregulation in this ND. Further, NSun2 expression was considerably lower in patients with AD aged <65 years [62]. NSun2 mediates tRNA methylation and reduces the susceptibility of these tRNAs to ANG-mediated cleavage [64]. These findings indicate that decreased NSun2 levels in patients with AD may compromise tRNA methylation, thereby enhancing ANG-mediated tRF generation. The above examples indicate that the dysregulation of tsRNAs is an important mechanism for the occurrence and development of AD.

### 4.2. tsRNAs in Parkinson’s disease (PD)

PD is a prevalent form of chronic progressive dyskinesia resulting from dopaminergic neuron depletion and α-synuclein Lewy body deposition within the substantia nigra. PD is the second most prevalent ND among the elderly [65]. The loss of nigral dopaminergic neurons results in symptoms, such as bradykinesia, tremors, and postural instability. Recent research has indicated that tsRNAs are involved in the development of PD. For example, 37-39 tRNA halves and ANG are potential regulators of PD in experimental systems [66, 67]. Zhang et al. reported that AS-tDR-011775 interacted with Mobp and influenced axon morphology. Further, they demonstrated that AS-tDR-005058 interacted with Erc1, and the interactions between the Rab6ip2/CAST2/ERC1/ELKS axis and proteins of the presynaptic active zone regulated neurotransmitter release [61]. Recent evidence suggests that the patterns of expression for tRFs in the cerebrospinal fluid, prefrontal cortex, and serum samples differ between patients with PD and healthy individuals, highlighting the potential of using these biomolecules as sensitive and specific biomarkers of PD [68]. Similarly, RNA-seq datasets have revealed differential patterns of tRF expressions in the prefrontal cortex, serum, and cerebrospinal fluid between control individuals and patients with PD that permit the stratification of these patients with excellent sensitivity and specificity. Essentially, tRF signatures may be harnessed as valuable non-invasive biomarkers of PD [66], although more work is required to establish the specific endonucleases that underpin disease-related factors. Indeed, patterns of tsRNA dysregulation have been reported in the context of PD [69, 70] and other NDs such as amyotrophic lateral sclerosis (ALS) [71]. Over 40 ANG mutations have been linked to PD in studies conducted over the past two decades[69, 72]. These mutations primarily affect ANG ribonuclease activity, thereby influencing tiRNA production. The ability of ANG to mediate neuroprotection is dependent on RNase activity, suggesting that tiRNAs may be involved in PD pathogenesis. For example, the production of tiRNA^Ala^- and tiRNA^Cys^-derived tiRNA-5s in an ANG-dependent fashion protects motor neurons under stress and inhibits translation and SG formation, underscoring the possible roles of these tiRNAs in NDs [37, 73]. Further, certain ALS-associated ANG mutations have been discerned in patients suffering from PD [69], and most ALS-related loss-of-function mutations affect RNase activity, suggesting a link between tiRNA biogenesis and motor neuron survival [74, 75]. Many tsRNAs perform substantial tasks in the pathogenesis and development of PD. Therefore, dysregulated tsRNAs should be further investigated.

### 4.3. tsRNAs in amyotrophic lateral sclerosis (ALS)

ALS is a serious progressive ND for which effective treatments are lacking. Patients with ALS suffer from progressive muscle and limb paralysis, ultimately compromising functions, including speech, swallowing, and respiration, due to spontaneous motor neuron degeneration [76]. As discussed above, ANG mutations that compromise the RNase activity of this enzyme are associated with ALS pathogenesis [71, 77] and reduced generation of ANG-derived tiRNAs that are important for suppressing protein synthesis [35], forming SGs [78], and inhibiting cytochrome c-mediated apoptosis [51]. While the mechanistic basis for ALS-associated neurodegeneration remains incompletely understood, motor neuron degeneration in affected patients is thought to occur as a consequence of apoptotic mechanisms [79]. The capacity of tiRNAs to exert anti-apoptotic effects and prevent neuronal death may at least partially underpin the relationship between ANG loss-of-function mutations and ALS incidence. Early studies on the role of tiRNAs in ALS revealed their ability to preserve motor neuron integrity. The loss of these tiRNAs or interactions between tiRNAs and pathogenic repetitive RNA species is thought to contribute to ALS pathogenesis [37]. ANG-dependent 5’-tiRNA^Ala^ and 5’-tiRNA^Cys^ create the structures of G-quadruplexes that enter motor neurons in humans, thereby protecting these cells in a YB-1-dependent fashion [37]. Furthermore, a recently published article explained that the fragment of 5’Val CAC tRNA is considerably upregulated in ALS mouse models and patient serums, highlighting its potential utility as a prognostic marker for ALS [80]. Greenway et al. first established *ANG* as an ALS susceptibility gene in a study conducted in 2004 [72]. More recent research has demonstrated that ALS-related *ANG* mutations primarily affect the RNase activity of this enzyme [71]. These early findings prompted additional studies on the effects of ANG on neuronal survival, and the protective effects of this protein have been detected under different stress circumstances. For instance, ANG can protect motor neurons versus excitotoxic injuries in a PI-3-kinase/Akt kinase-dependent behavior [81]. Further, ANG improves the ability of neurons to tolerate ER stress, hypoxia, and trophic-factor withdrawal-induced death of cells, whereas ALS-related *ANG* mutant isoforms are not protective in these contexts [81, 82]. Saikia et al. reported that ANG protects primary neurons against hyperosmotic stress-induced cell death [51]. The role of tsRNAs in ALS is still very limited. A notable number of surveys have revealed that the changes in essential gene expression during the pathogenesis of ALS are correlated with different levels of tsRNAs in ALS patient’s serum. However, the detailed action mechanism of tsRNAs is still largely unclear.

### 4.4. tsRNAs in pontocerebellar hypoplasia (PCH)

Pontocerebellar hypoplasia (PCH) comprises a series of 13 subtypes of early-onset NDs characterized according to neuropathological, clinical, and MRI criteria [83, 84]. Patients with PCH often present with degeneration and limited development of the pons and cerebellum, indicating that this disease begins during the prenatal period. The genetic causes underlying many PCH cases remain poorly understood, and additional PCH subtypes will likely be identified in the future. However, prior research suggests that PCH often occurs due to the defects in essential cellular homeostatic processes, including tRNA synthesis and RNA metabolism [85]. The *CLP1* R140A mutation has been identified in PCH cases, and *CLP1*-deficient mice and zebrafish exhibit many of the same developmental and neuromuscular defects that are present in PCH patients [3, 56, 57]. This mutation results in mature tRNA depletion and unspliced pre-tRNA accumulation within neurons [56], in addition to driving linear intron accumulation [57]. When a precise comparison with the 5’-exon transfection that does not alter cell survival, was made, transfection of neurons with the 5’- unphosphorylated tRF associated with the 3’-exon of pre-tRNATyr, a CLP1 substrate, impaired the ability of these cells to survive under oxidative stress [56]. Mice lacking CLP1 kinase activity exhibit 3’ leader exon tRF accumulation and consequent sensitization of cells to oxidative stress, driving p53-dependent cell death [3]. Further, mutations in tRNA splicing endonuclease complex genes, including TSEN54, TSEN34, TSEN15, and TSEN2, have been observed in patients with type 4 and 2 PCH that exhibit structural defects and poor cerebellar development[86]. Although additional investigations are necessary to fully clarify the association between CLP1 activity, tRNA splicing, and tRFs in PCH, these results collectively suggest that dysregulated tRNA metabolism is associated with neurodegenerative processes in PCH.

### 4.5. Potential clinical and therapeutic applications of tsRNAs in NDs

As discussed above, various tsRNAs serve as vital regulators of CNS development and function, and the dysregulation of tsRNA signaling networks may lead to pathological outcomes. Investigating the mechanisms by which tsRNAs regulate cellular processes may result in detecting novel diagnostic biomarkers and/or therapeutic targets for NDs. Indeed, tsRNAs themselves represent viable targets as antagonistic sequences for RNAs, which may be generated and tested for selective disease treatments in an individualized manner. Advancements in the technologies of RNA-seq and associated bioinformatics tools have led to a better appreciation of the key roles of tsRNAs in various NDs. The tissue-specific expression patterns of tsRNAs highlight the potential utility of these biomolecules as diagnostic or prognostic biomarkers that may aid in drug development. Considering the high specificity of tsRNAs, it may be feasible to design novel therapeutics that target these non-coding RNAs with minimal off-target effects. Nevertheless, most studies have only detected differences in tsRNA expression in the context of NDs, while the functional roles of these non-coding RNAs in disease pathogenesis remain poorly understood. The limited amount of information encoded within tsRNAs and the limitations inherent to studies of complex neuro-degenerative conditions have constrained efforts to elucidate the regulatory roles of these biomolecules in disease progression. Further in-depth research on the microenvironmental impact of these tsRNAs is essential. Indeed, past findings underscore the clinical value of further research in this domain. For example, transfecting patient-derived neurons with specific tRF-5s reduces cell survival in an *in vitro* model of oxidative stress [56]. Moreover, Ivanov et al. demonstrated that the DNA-based 5’tiDNAAla enters human motor neurons by forming a G-quadruplex structure, thereby protecting these cells against stress induced cell death and underscoring the potential therapeutic utility of aptamers that mimic tiRNAs [37]. Essentially, tiRNAs may be useful as both therapeutic tools and treatment targets for NDs.

Concerning the potential diagnostic perspective of tsRNAs in neurodegenerative diseases, the following aspects must be considered. First, as a novel diagnostic and potential therapeutic marker, tsRNAs should be easily acquired and measured in the clinics. The higher expression stability presented by tsRNAs make them suitable as diagnostic biomarkers. As mentioned above, Magee et al. found tRFs from prefrontal cortex, CSF, and serum those could differentiate PD patients from controls and may serve as reliable biomarkers for PD[68]. Consequently, they could potentially be non-invasive biomarkers for PD in the future. Second, further investigations are necessary to distinctly identify the roles of tsRNAs in NDs. Finally, the mechanisms responsible for the therapeutic effects of tsRNAs in NDs should be well characterized before their clinical application. Nevertheless, we remain quite confident that this challenge will be solved in the future.

In terms of the therapeutic methods of tsRNAs in NDs, recent developments have suggested that tsRNAs can potentially be developed as biopharmaceuticals. However, the development of tsRNAs as biopharmaceuticals poses many challenges, including their function, mechanism, production, purification, and effective delivery into the brain, which need to be addressed in future clinical trials. Thus, *in vivo* and *in vitro* studies should be performed prudently before clinical trials. Overexpression or knockout techniques can be used to confirm target tsRNA functions in disease development *in vivo*. Subsequently, genome-editing techniques such as CRISPR/Cas9 can be used to investigate the favorable effects and side effects of target tsRNA treatment in vitro. Third, AAV vectors or other harmless viral vectors could be utilized for target tsRNA treatment in a clinical trial. Finally, the brain has its own particular issues, such as the blood-brain-barrier penetration and off-target effects that should be taken seriously. However, we believe that with these improvements, it will be possible to use tsRNA as a novel therapeutic target for NDs in the near future.

### 4.4. Current challenges and further direction

Many dysregulated tsRNAs causing degenerative diseases were discovered with the application of high-throughput sequencing technology. However, there are still several challenges in tsRNAs research. First, the potential functions, underlying molecular mechanisms, biogenesis, and classification of tsRNAs in NDs need to be imminently clarified. Although the dysregulation of several tsRNAs has been reported in NDs, their mechanisms and function are elusive. Research into tsRNAs as neurodegenerative biomarkers is still in its infancy, and these are far from being used as promising diagnostic and prognostic molecular biomarkers or therapeutic targets in the clinics. Second, there is an urgent need to identify and quantify tsRNAs correctly. High-throughput sequencing, microarrays, quantitative reverse transcription-polymerase chain reaction, northern blotting, and bioinformatics are currently used for screening and detecting certain tsRNAs. Furthermore, a more efficient combination method for better quantifying tsRNAs sensitively and specifically needs to be developed. Third, the detailed relationship between tsRNAs and miRNAs has not been fully characterized.

tsRNAs have a wide range of roles in the normal development and function of the CNS. They are key modulators of gene expression and are involved in several neuroprotective mechanisms. Indeed, it is well-established that the dysregulation of tsRNAs ultimately leads to the genesis and development of various NDs. tsRNAs involved in the pathogenesis of several NDs presents a novel opportunity for elucidating previously unrecognized underlying molecular mechanisms as well as for these molecules to be explored as potential diagnostic biomarkers and therapeutic targets in NDs. Further study in the patterns and profiling of tsRNA expression should result in the discovery of many more novel targets and biomarkers in NDs. Further in-depth investigation to elucidate the functions of tsRNAs in RNA-mediated gene regulation is likely to remain an active and intense area of research.

In summary, tsRNAs have appeared as essential regulators of diverse physiological processes and hold a substantial promise for the diagnosis and treatment of many NDs. Future efforts to harness tsRNAs may lead to improvements in the quality of life of individuals affected by these conditions.
